# Combating virulence of Gram-negative bacilli by OmpA inhibition

**DOI:** 10.1038/s41598-017-14972-y

**Published:** 2017-10-31

**Authors:** Xavier Vila-Farrés, Raquel Parra-Millán, Viviana Sánchez-Encinales, Monica Varese, Rafael Ayerbe-Algaba, Nuria Bayó, Salvador Guardiola, María Eugenia Pachón-Ibáñez, Martin Kotev, Jesús García, Meritxell Teixidó, Jordi Vila, Jerónimo Pachón, Ernest Giralt, Younes Smani

**Affiliations:** 1 Institute for Research in Biomedicine (IRB Barcelona), Barcelona Institute for Science and Technology (BIST), Barcelona, Spain; 2 Institute of Biomedicine of Seville (IBiS), University Hospital Virgen del Rocío/CSIC/University of Seville, Seville, Spain; 3 Barcelona Centre for International Health Research (CRESIB, Hospital Clínic-Universitat de Barcelona), Barcelona, Spain; 4 Faculty of Chemistry, University of Barcelona, Barcelona, Spain

## Abstract

Preventing the adhesion of pathogens to host cells provides an innovative approach to tackling multidrug-resistant bacteria. In this regard, the identification of outer membrane protein A (OmpA) as a key bacterial virulence factor has been a major breakthrough. The use of virtual screening helped us to identify a cyclic hexapeptide AOA-2 that inhibits the adhesion of *Acinetobacter baumannii*, *Pseudomonas aeruginosa* and *Escherichia coli* to host cells and the formation of biofilm, thereby preventing the development of infection *in vitro* and in a murine sepsis peritoneal model. Inhibition of OmpA offers a strategy as monotherapy to address the urgent need for treatments for infections caused by Gram-negative bacilli.

## Introduction

Treatment of bacterial infections, especially those caused by strains resistant to all known antibiotics, is a major concern. The number of antibiotics approved by the FDA cannot keep pace with the rapid development of antimicrobial resistance. There is, therefore, an urgent need to find new antibiotics against extensive- (XDR) and pandrug-resistant (PDR) Gram-negative bacilli (GNB)^1^. Majority of the antibiotics currently in use are either bactericidal or bacteriostatic and most work against a broad spectrum of bacteria. Two key approaches can help alleviate the problem of antibiotic resistance, first the development of single-pathogen therapies focused on the specific treatment of infections caused by a single pathogen highly resistant to antimicrobial agents^2^, and second targeting bacterial virulence factors without inhibiting bacterial growth, which can slow the development of drug resistance by reducing the selective pressure on the bacteria^3,4^.

Many bacterial pathogens use their outer membrane proteins (OMPs) to interact with the host environment in order to induce the expression of virulence factors, to invade tissues, and to escape the immune system^4–6^. These pathogens appear to use the outer membrane protein A (OmpA), among others, to attach to host cells and to mediate bacterial entry^7^. OmpA is a beta-barrel porin that is highly conserved among bacterial species, especially throughout GNB^7^. This protein is multifunctional, with a variety of *in vitro* and *in vivo* biological properties of interest. It has been shown to be involved in adherence to epithelial cells^8–10^, translocation into epithelial cells nucleus^11^, induction of epithelial cell death and mouse mortality^12–14^, biofilm formation^9,15^, and binding to factor H postulated to allow bacteria to develop serum-resistance^15–18^. In humans this protein has been recently associated with the development of pneumonia and bacteremia by a GNB, *Acinetobacter baumannii* ^14^.

Given the prominence of OmpA in promoting the GNB virulence, we designed a series of OmpA inhibitors and tested their effectiveness *in vitro* and *in vivo* in preventing infection by the most prevalent GNB in clinical settings as *A. baumannii*, *Pseudomonas aeruginosa* and *Escherichia coli*.

## Results & Discussion

### Peptide design and synthesis

OmpA of *E. coli* is the best-characterized member of a large family of homologous bacterial proteins. The three-dimensional structure of the two independently folded domains of *E. coli* OmpA has been determined by X-ray and NMR. The 170 N-terminal residues form an eight-stranded β-barrel that is embedded in the outer membrane^19–22^. The globular structure of the periplasmic C-terminal part belongs to the OmpA-like domain-fold family^23^.

We performed an initial computational analysis with the SiteMap module of Schrodinger Suite^24^ and identified a potentially druggable area for ligand binding in a cavity formed by the extracellular loops of the N-terminal transmembrane (TM) domain of OmpA. We hypothesized that the size and shape of this predicted druggable area of OmpA may allow for accommodation of a hexapeptide ligand. Considering that cyclization increases the proteolytic resistance and restraints the conformational flexibility of peptide sequences, we designed a virtual library of 26 C2-symmetric cyclic hexapeptides as potential OmpA-binders (Table 1). All cyclic hexapeptides contained two fixed proline residues to facilitate cyclization. This peptide library was subsequently computationally screened against the TM domain of OmpA of both *E. coli* and *A. baumannii* and ranked on the basis of the scoring function (see “Methods” section). As no three-dimensional structure of the TM domain of *A. baumannii* OmpA was found in the Protein Data Bank (PDB), a homology model using the i-TASSER server was built using the structures of the homologous domain of *E. coli* (PDB entries 1G90, 2GE4, 1QJP, and 1BXW) as template. Similar docking results were obtained for the OmpA proteins of both pathogens. Notably, all peptides containing tryptophan (Trp) and arginine (Arg) residues showed better *in silico* activity compared to other hexapeptides (Table 1). The peptide-protein complex models generated by docking suggested that the best-scoring peptides bind in the same protein region and adopt similar orientations in the interaction with both proteins (Fig. S1). One of the Trp residues of the peptide acts as an anchor and inserts in the border of the β-barrel, while the remaining peptide residues contact with the extracellular flexible loops. The possible contact of the peptide with extracellular loops could affect the function of OmpA by reducing their interaction with host cells and consequently bacterial pathogenesis. It is well known that inhibition and mutation of the extracellular loops of OmpA affect the pathogenesis and the interaction of *E. coli* with host cells^25,26^.

**Table 1:** Docking score of the virtual library of C2-symmetric cyclic hexapeptides.

Cyclic peptide	Docking score
&Arg-D-Pro-Trp-Arg-D-Pro-Trp&	−10.8
&Trp-D-Pro-Arg-Trp-D-Pro-Arg&	−10.6
&Arg-Pro-Trp-Arg-Pro-Trp&	−10.3
&Trp-Pro-Arg-Trp-Pro-Arg&	−10.0
&Trp-Pro-D-Arg-Trp-Pro-D-Arg&	−9.2
&Arg-Pro-D-Trp-Arg-Pro-D-Trp&	−8.9
&Arg-D-Pro-D-Ile-Arg-D-Pro-D-Ile&	−8.5
& Trp- D-Pro-Arg-Trp-D-Pro-Arg&	−8.5
&Arg-D-Pro-Ile-Arg-D-Pro-Ile&	−8.2
&Ser-D-Pro-Trp-Ser-D-Pro-Trp&	−7.5
&Ser-D-Pro-D-Trp-Ser-D-Pro-D-Trp&	−7.4
&Ser-D-Pro-D-Ile-Ser-D-Pro-D-Ile&	−7.3
&Ser-D-Pro-Ile-Ser-D-Pro-Ile&	−7.2
&D-Ser-Pro-Ala-D-Ser-Pro-Ala&	−7.1
&Glu-D-Pro-Trp-Glu-D-Pro-Trp&	−7.0
&Ile-D-Pro-D-Trp-Ile-D-Pro-D-Trp&	−6.8
&Ser-D-Pro-Ala-Ser-D-Pro-Ala&	−6.8
&Ile-D-Pro-Trp-Ile-D-Pro-Trp&	−6.7
&D-Arg-Pro-Trp-D-Arg-Pro-Trp&	−6.1
&Ser-D-Pro-Glu-Ser-D-Pro-Glu&	−6.1
&Glu-D-Pro-D-Trp-Glu-D-Pro-D-Trp&	−6.1
&Ser-D-Pro-D-Glu-Ser-D-Pro-D-Glu&	−6.1
&Ser-Pro-Ala-Ser-Pro-Ala&	−5.6
&Glu-D-Pro-Ile-Glu-D-Pro-Ile&	−4.6
&Glu-D-Pro-D-Ile-Glu-D-Pro-D-Ile&	−4.4
&Gly-Pro-Ala-Gly-Pro-Ala&	−2.9

& symbol means cyclic peptides

To experimentally validate the virtual screening, we synthesized a set of seven peptides (Table 2, Fig. S2). Four of them (AOA-1 to AOA-4) were Arg-Trp-containing peptides in which the position and/or stereochemistry of the amino acid residues were modified. These included the best-scoring docking peptides (AOA-1 and AOA-2). Additionally, two peptides were synthesized in which the positively charged arginine residue was replaced by a neutral hydrophilic serine (AOA-5) or by a negatively charged glutamic acid (AOA-6). As a control, the linear version (SXV4) of the *in silico* most active peptide was also synthesized.

**Table 2:** List of synthesized hexapeptides based on the computationally screened library.

Name	Cyclic peptide	Purity (%)	Rational
AOA-1	&Arg-D-Pro-Trp-Arg-D-Pro-Trp&	96	Top-scoring docking peptide
AOA-2	&Trp-D-Pro-Arg-Trp-D-Pro-Arg&	92	Top-scoring docking peptide
AOA-3	&Arg-Pro-D-Trp-Arg-Pro-D-Trp&	100	Different stereoisomer
AOA-4	&D-Arg-Pro-Trp-D-Arg-Pro-Trp&	95	Different stereosiomer
AOA-5	&Ser-D-Pro-Trp-Ser-D-Pro-Trp&	100	Arg replaced by Ser
AOA-6	&Glu-D-Pro-Trp-Glu-D-Pro-Trp&	95	Arg replaced by Glu
SXV4	Ac-Trp-D-Pro-Arg-Trp-D-Pro-Arg-OH	100	Negative Control

### *In vitro* effect of OmpA inhibitor peptides on *A. baumannii* growth and contact with host cells

In the initial screening, the bactericidal activity of the synthetic peptides and their toxicity on human lung epithelial cells (A549) were evaluated. We confirmed that these peptides did not show bactericidal or cytotoxic activities, compared with colistin, as the positive control peptide (Tables S1 and S2), and therefore fulfill all the features to be only potential blockers of OmpA, without killing either bacteria or cells.

The ability to reduce *A. baumannii* adherence to A549 cells was tested in all the compounds synthesized. Among them, peptide AOA-2 (&Trp-D-Pro-Arg-Trp-D-Pro-Arg&) presented the greatest ability to reduce the *A. baumannii* adherence to A549 cells (Fig. S3).

### NMR detection of AOA-2 binding to OmpA

Considering that AOA-2 provided analogous docking results with the *E. coli* and *A. baumannii* OmpA homologs, we hypothesized that *E. coli* OmpA represents an optimal model system for *in vitro* characterization of the intermolecular interaction of AOA-2 with OmpA proteins. *E. coli* OmpA has been extensively characterized and the protocol to produce pure and functional *E. coli* OmpA has been previously reported^21^.

**Figure 1:**
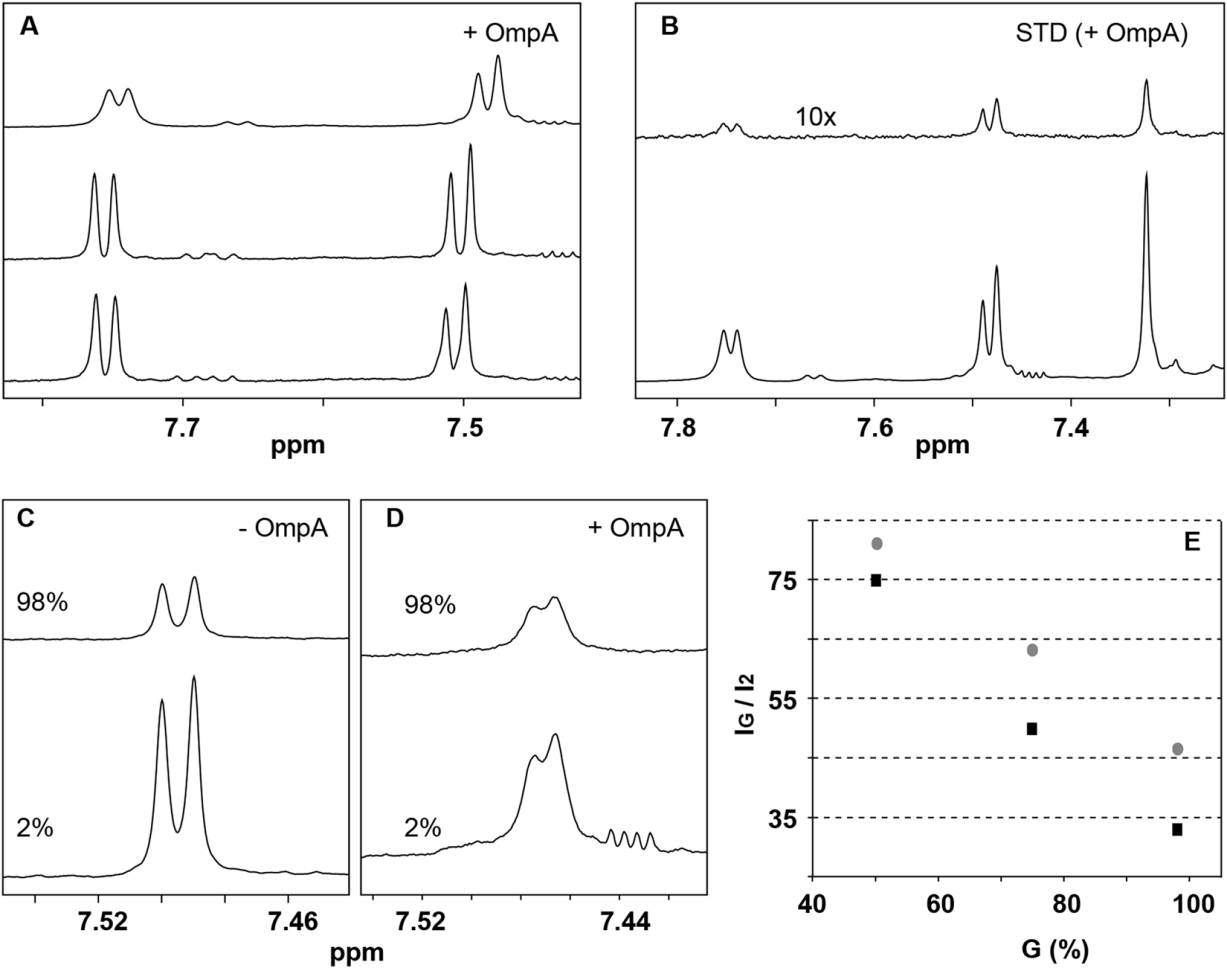
NMR detection of AOA-2 binding to OmpA. (**A**) Aromatic region of the ^1^H-NMR spectra of AOA-2 (750 μM) (bottom), AOA-2 (750 μM) in the presence of 1 mM Triton X-100 (middle), and AOA-2 (750 μM) in the presence of 1 mM Triton X-100 and 8 μM OmpA (top). The presence of OmpA causes signal broadening and chemical shift changes in the AOA-2 resonances. (**B**) Selected region of the STD spectrum (top) of AOA-2 (750 μM) in the presence of OmpA (8 μM). The reference off-resonance spectrum (bottom) is also shown. The STD spectrum has been scaled by a factor of 10. (**C**–**E**) Diffusion NMR detection of AOA-2 binding to OmpA. (**C**,**D**) Expansions of PFG-NMR diffusion experiments of AOA-2 (750 μM) with 1 mM Triton X-100 in the absence (**C**) and in the presence of 8 μM OmpA (**D**). In both panels, diffusion gradients were 2% (bottom) and 98% (top) of their maximum value (≈55 G/cm). (**E**) Ratio of the intensity (100*I_G_/I_2%_) of the AOA-2 signal resonating at ca. 7.50 ppm for a given diffusion gradient strength (I_G_) with respect to that measured (I_2%_) with the weaker diffusion gradient (2%). Peak intensity ratios were obtained in the absence (black squares) and in the presence of 8 μM OmpA (grey circles). As an indication of binding, AOA-2 appeared to diffuse slower (smaller signal attenuation) in the presence of OmpA than in isolation.

We assessed the binding of AOA-2 to *E. coli* OmpA by solution NMR. As an indication of binding, the presence of OmpA solubilized in 1 mM Triton X-100 causes signal broadening and chemical shift changes in the proton resonances of AOA-2. The 1D ^1^H NMR spectrum of AOA-2 was unaltered by the presence of 1 mM Triton X-100, demonstrating that the perturbations were induced by OmpA (Fig. 1A).

The peptide-protein interaction was also clearly observable by Saturation Transfer Difference (STD) NMR experiments of AOA-2 (750 μM) in the presence of OmpA (8 μM). The resulting STD spectrum was obtained by subtracting a spectrum with irradiation at −0.75 ppm, which selectively saturated the protein, from an analogous spectrum with irradiation at 50 ppm, which did not perturb any AOA-2 or OmpA resonances. The STD spectrum reveals signals of the binding peptide that have been perturbed by saturation transfer from the protein (Fig. 1B).

Binding was further confirmed by pulsed field gradient (PFG) NMR-based diffusion experiments. In these experiments, PFG causes a decrease in signal intensity that depends on the gradient strength, and on the translational diffusion of the observed molecule, and is more pronounced for rapidly diffusing, small molecules. As shown in Fig. 1C–E, the attenuation of AOA-2 signals was smaller in the presence of the protein indicating that AOA-2 is interacting with OmpA and then has a slower translational diffusion.

### *In vitro* effect of AOA-2 against GNB contact with host cells

**Figure 2:**
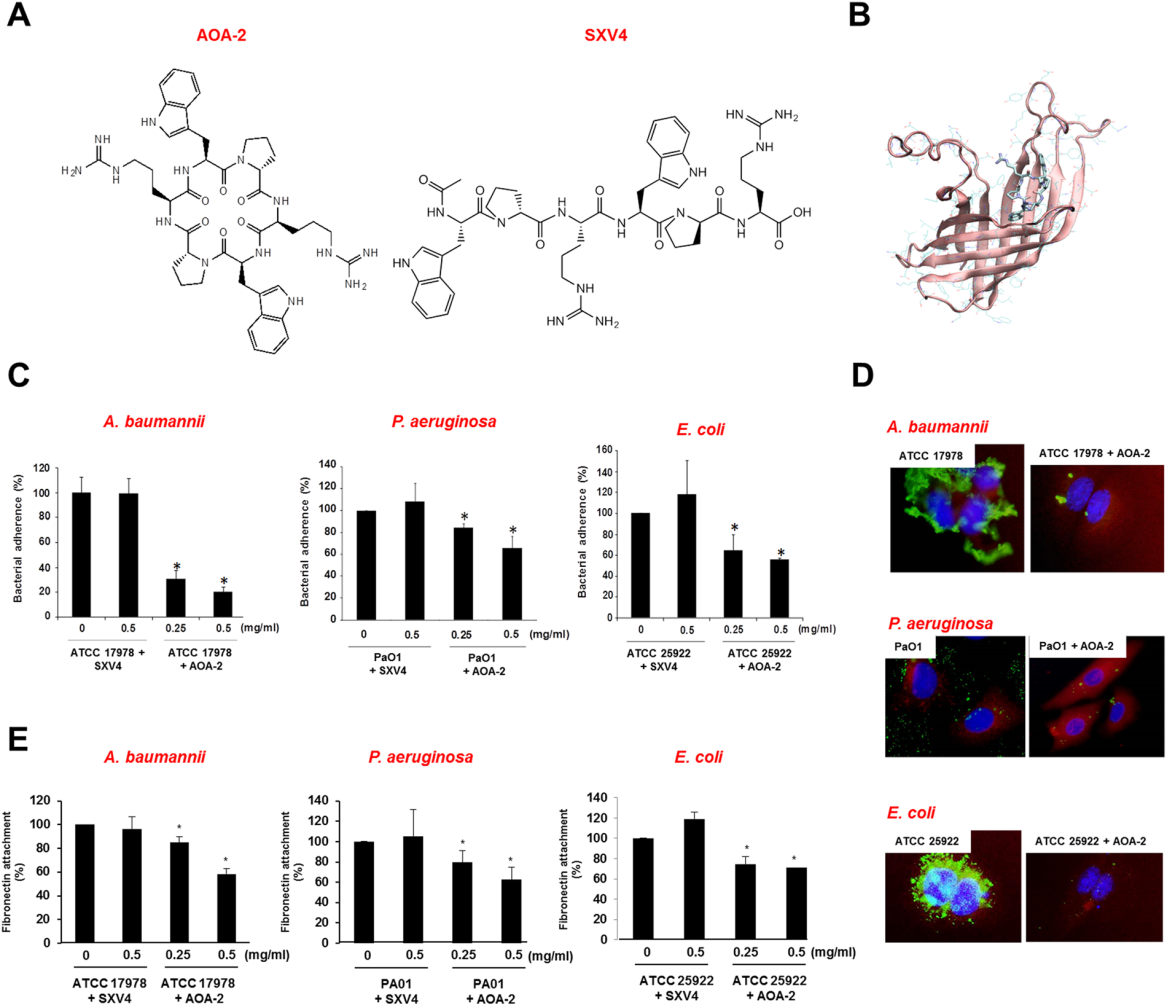
AOA-2 inhibits *A. baumannii*, *P. aeruginosa* and *E. coli* contact with host cells *in vitro*. (**A**) Chemical structure of AOA-2 and SXV4 (negative control lineal peptide). (**B**) Three-dimensional representation of AOA-2 and OmpA complex. (**C**) Adhesion of *A. baumannii* ATCC 17978, *P. aeruginosa* PaO1, and *E. coli* ATCC 25922 strains to A549 cells. A549 cells were infected with 10^8^ CFU/mL of ATCC 17978, PaO1 or ATCC 25922 strains for 2 h in the presence of AOA-2 or SXV4 (0, 0.25 or 0.5 mg/mL). (**D**) Immunostaining of fibronectin of A549 cells (red) and ATCC 17978, PaO1, and ATCC 25922 strains (green) pretreated with AOA-2 (0 and 0.25 mg/mL), after bacterial adherence for 2 h, was performed by specific primary antibodies against these strains and their respective secondary antibodies. Blue staining shows the location of A549 cell nuclei. (**E**) Bacterial interaction with immobilized fibronectin. ATCC 17978, PaO1, or ATCC 25922 strains pretreated with AOA-2 or SXV4 (0, 0.25 or 0.5 mg/mL) were incubated in a fibronectin-coated 96-well plate for 2 h. Bacteria adhered to fibronectin were quantified by serial dilutions as described in materials and methods. Representative results of three independent experiments are shown; data are means ± SEM. *P < 0.05: between untreated and treated groups.

AOA-2 (Fig. 2A,B) was selected for the following studies on the basis of its greater ability to reduce the bacterial adherence (Fig. S3). This peptide showed a reduction in the adherence of *A. baumannii* to A549 cells by more than 60% *in vitro* using 0.25 mg/mL of the compound (Fig. 2C). Due to highly conservation of OmpA among most of the GNB, the same assay using *P. aeruginosa* and *E. coli* was performed also observing a significant reduction in adherence (Fig. 2C), and without presenting bactericidal activities even at the highest AOA-2 concentration tested (Fig. S4). It is important to note that AOA-2 at concentrations (0.25–1 mg/mL) presents different bacteriostatic activities on different bacteria. Thus, it appears to have high anti-adherence/poor bacteriostatic activity on *A. baumannii*, and low anti-adherence/relevant bacteriostatic activity on *P. aeruginosa*, with an intermediate behaviour in the case of *E. coli*.

We previously reported that *ompA-*deficient *A. baumannii* is less adherent to abiotic surfaces and disseminate less between murine organs^14,27^ and that recombinant OmpA shows high affinity for fibronectin, an extracellular matrix protein^28^. Due to this fact, assays to test the activity of this peptide blocking the interaction between fibronectin and bacteria (*A. baumannii, P. aeruginosa* and *E. coli*) were performed, and the results showed a significant decrease in the bacterial adherence to fibronectin after treatment with AOA-2 (Fig. 2D,E). In the same line, other approach using D-amino acids has been performed recently to assess their antivirulence activity against *A. baumannii* and *P. aeruginosa* ^29^, and showed that some of them can inhibit both pathogens adherence to eukaryotic cells and protect against infection of these eukaryotic cells with *P. aeruginosa* ^29^.

### AOA-2 effect on biofilm formation by GNB

Many reports have demonstrated the involvement of OmpA in biofilm formation by GNB^10,27,30,31^. These reports showed that *ompA-*deficient *A. baumannii* and *E. coli* did not produce biofilm^10,27,31^. Here, we showed that treatment with AOA-2 diminishes the biofilm formation of all the reference strains and clinical isolates of *A. baumannii*, *P. aeruginosa*, and *E. coli* (Table 3). Bacterial biofilms have significant impacts in industrial and clinical settings and there is therefore an urgent need to develop new compounds to prevent biofilm formation. Our data with AOA-2 showed remarkable results and are consistent with those previously reports for other inhibitors^29,32,33^.

**Table 3:** Effect of AOA-2 on biofilm formation by reference strains and clinical isolates of *A. baumannii*, *P. aeruginosa* and *E. coli*.

Pathogen	Strain	Absorbance at 580 nm	
		Without AOA-2	With AOA-2
***A. baumannii***	ATCC 17978	1.16 ± 0.14	0.54 ± 0.23
	ATCC 19606	0.98 ± 0.1	0.61 ± 0.1
	77	0.98 ± 0.05	0.73 ± 0.08
	C4	1.04 ± 0.003	0.5 ± 0.006
	C5	0.9 ± 0.05	0.42 ± 0.06
	C12	0.99 ± 0.003	0.51 ± 0.02
	IB1	1.12 ± 0.11	0.58 ± 0.04
	IB2	1.08 ± 0.02	0.43 ± 0.06
	HC1	0.83 ± 0.06	0.41 ± 0.1
	HC2	0.97 ± 0.11	0.52 ± 0.1
***P. aeruginosa***	PaO1	0.77 ± 0.02	0.53 ± 0.05
	15	0.86 ± 0.02	0.2 ± 0.01
	17	0.84 ± 0.02	0.18 ± 0.002
	61	0.57 ± 0.01	0.21 ± 0.01
	127	0.71 ± 0.02	0.18 ± 0.01
	142	0.93 ± 0.03	0.27 ± 0.01
	160	0.91 ± 0.02	0.17 ± 0.01
	184	1.00 ± 0.12	0.41 ± 0.07
	204	0.71 ± 0.02	0.37 ± 0.01
***E. coli***	ATCC 10536	0.68 ± 0.019	0.2 ± 0.02
	12–69	0.41 ± 0.02	0.19 ± 0.02
	7–9	0.27 ± 0.03	0.09 ± 0.01
	7–2	0.26 ± 0.03	0.13 ± 0.01
	11–51–2	0.32 ± 0.01	0.21 ± 0.007
	5–38	0.17 ± 0.01	0.09 ± 0.006
	12–74	0.23 ± 0.01	0.18 ± 0.007

### *In vitro* and *in vivo* effect of AOA-2 against GNB virulence

**Figure 3:**
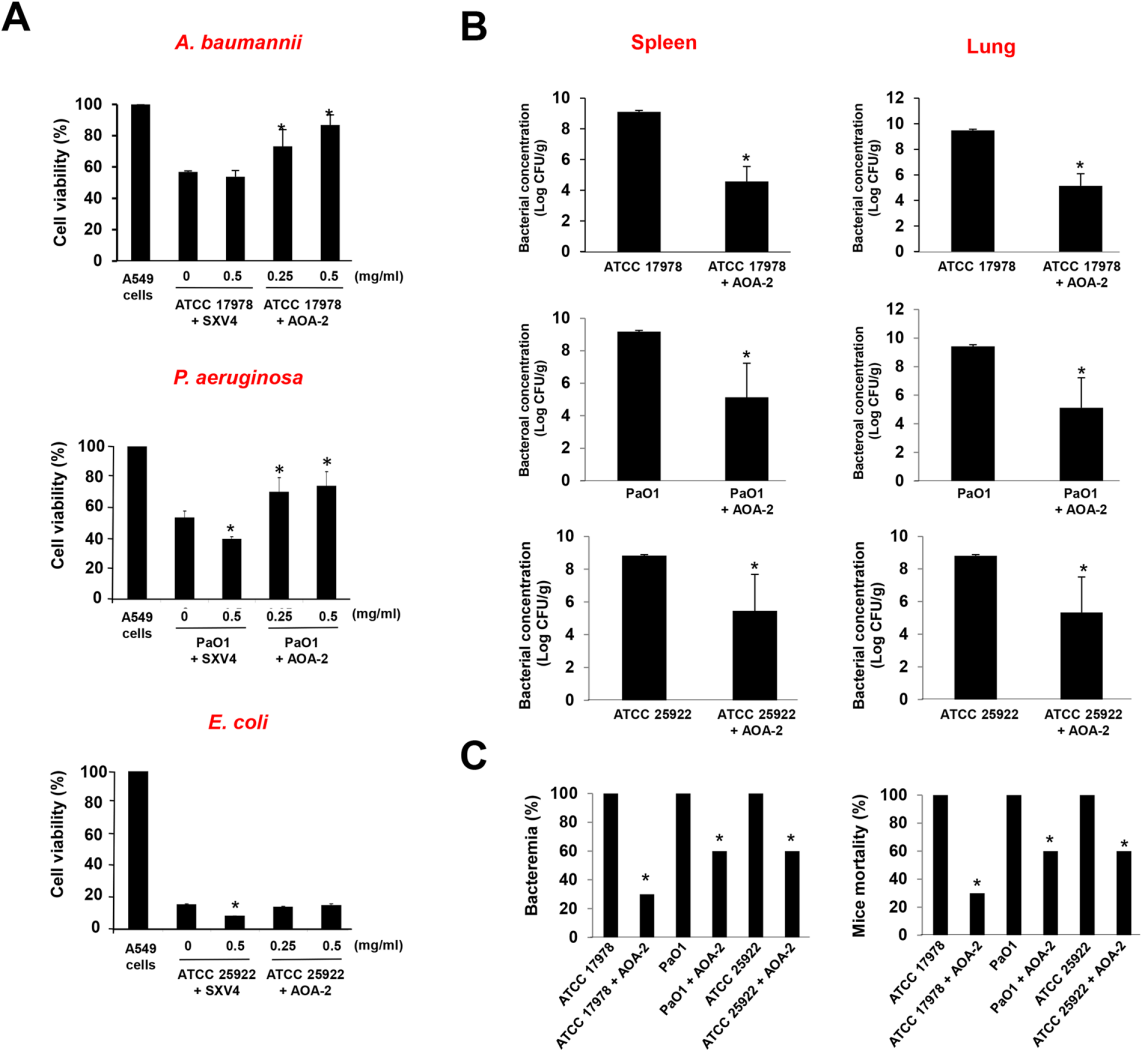
AOA-2 inhibits *A. baumannii*, *P. aeruginosa* and *E. coli* virulence *in vitro* and *in vivo*. (**A**) Cell death induced by *A. baumannii* ATCC 17978, *P. aeruginosa* PaO1, and *E. coli* ATCC 25922 strains. A549 cells were infected for 24 h with 10^8^ CFU/mL of ATCC 17978, PaO1 or ATCC 25922 strains pretreated with AOA-2 or SXV4 (negative control lineal peptide) (0, 0.25 or 0.5 mg/mL). Bacterial cytotoxicity was assessed by monitoring the mitochondrial reduction activity using the MTT assay. Representative results of three independent experiments are shown; data are means ± SEM. (**B**) CFU/g of ATCC 17978, PaO1 or ATCC 25922 strains harvested from spleen and lungs of mice infected intraperitoneally with the MLD of ATCC 17978, PaO1 or ATCC 25922 strains and treated or not with AOA-2 (10 mg/kg/d, for 3 days) 2 h after bacterial inoculation. (**C**) Bacteremia and survival from the previous mouse groups. *P < 0.05: between untreated and treated groups.

We have proved using different assays that AOA-2 is able to block the interaction between different bacteria and host cells, however the real aim of this compound is to prevent the cell death. The presence of 0.25 or 0.5 mg/mL AOA-2, prevented cell death dependent on bacterial adherence^10,34^, with the exception of *E. coli* strain ATCC 25922 (Fig. 3A). Interestingly, AOA-2 has no effect on cell death caused by *ompA*-deficient *A. baumannii* which confirm that AOA-2 specifically acts by inhibiting OmpA function (Fig. S5). The failure of AOA-2 against *E. coli* may be attributable to the presence of other highly virulent factors circumventing the loss of OmpA^35^, acting differently to those of *P. aeruginosa* and *A. baumannii*. The difference in activity between *A. baumannii* and the other two bacterial strains is due to the fact that the compound was designed specifically to be active against *A. baumannii* OmpA; however, it is also active against other GNBs due to the high sequence homology of the OmpA protein, therefore we may think that better results could be achieved for both *E. coli* and *P. aeruginosa*, by using its own OmpA or homologous protein.

We have demonstrated that AOA-2 reduced the interaction of GNB with host cells and prevented their death *in vitro*, and further studies in mice did not reveal any evidence of toxicity at a dose ≤40 mg/kg (Table S3). To prove the bioavailability of AOA-2 when administered i.p. to mice pharmacokinetic studies indicated that AOA-2 showed good activity (Fig. S6).

In a peritoneal sepsis murine model, administration of 10 mg/kg AOA-2 to mice 2 h after intraperitoneal (i.p.) challenge with minimal lethal doses of *A. baumannii* ATCC 17978, *P. aeruginosa* PaO1, and *E. coli* ATCC 25922 strains reduced the bacterial load in the spleen (4.54, 4.04, and 3.36 log CFU/g, respectively) and lungs (4.34, 4.29, and 3.47 log CFU/g, respectively) (Fig. 3B). In addition to this significant decrease of the bacterial load positive blood cultures and mouse mortality were also decreased (Fig. 3C). The possible explanation of the good therapeutic effect of AOA-2 could be the fact that AOA-2 would affect the interaction of bacteria with host cells which prevents their evasion from the immune host defence. These results are consistent with previous observations that absence of OmpA in *A. baumannii* and *E. coli* is associated with the loss of their virulence *in vivo* by reducing their dissemination between organs and animals mortality^14,15^.

So far, we have reported a cyclic hexapeptide (AOA-2) able to inhibit the GNB adherence to host cells and, consequently, an increase in the cell viability. At the same time, this peptide shows a potent inhibitor activity on the biofilm formation by these GNB. AOA-2 *in vivo* administration shows a large decrease in the bacterial loading in both spleen and lungs together with a significant decrease in mouse mortality, especially in *A. baumannii*.

In summary, this drug discovery program must be considered as an initial stage of the development of a novel class of antimicrobial agents. The efficacy of AOA-2 can be further tested in experiments administering this new peptide in combination with antimicrobial agents used in the clinical setting, with the aim to improve the therapeutic efficacy in severe infections, avoiding their mortalities despite the currently available antimicrobial therapies.

## Methods

### Bacterial strains

The list of bacterial strains was regrouped in Table S4.

### Docking and molecular modelling

All molecular docking calculations, and preparation of protein and ligand systems were done with SCHRODINGER software package^24^. Protein structures of the transmembrane domain of *A. baumannii* OmpA were obtained by homology modeling. Homology modeling constructs a model of the “target” protein from its amino acid sequence and an experimental three-dimensional structure (X-ray or NMR) of a related homologous protein (“template”). I-TASSER online server (http://zhanglab.ccmb.med.umich.edu/I-TASSER/) was used for homology modeling^36^. The amino acid sequence was taken from Genbank, and template structures used in the modeling were taken from the *E. coli*, OmpA transmembrane domain structures presented in the Protein Data Bank (PDB) depository. Two PDB entries obtained from NMR (1G90 and 2GE4), and two X-Ray crystals (1QJP, 1BXW) were used in all homology models. Best-ranked models were prepared with Protein Preparation Wizard, minimized^24^, and further relaxed including short (5 ns) explicit solvent molecular dynamics (MD) simulations with Desmond software^37^. The SiteMap module of the Schrodinger Suite^24^ identified a potentially druggable area for ligand binding in the border of opening of the transmembrane domain of OmpA and flexible extracellular loops. Same area was used for constructing docking grids for Glide. A library of cyclic hexapeptides was used for virtual screening. All peptide structures were built, optimized by LigPrep^24^ and additional MacroModel conformational search techniques were applied to all molecules. Thus, for each molecule of the library were obtained 10 to 20 different molecular conformations. All received conformations were docked using XP Glide protocol and best-scored results for each hexapeptide were stored.

### Materials for peptide synthesis

Protected amino acids and resins were supplied by Luxembourg Industries (Tel-Aviv, Israel), Neosystem (Strasbourg, France), Calbiochem-Novabiochem AG (Laüfelfingen, Switzerland), PolyPeptide Laboratories (Torrance, CA USA), Bachem AG (Bubendorf, Switzerland), and Iris Biotech (Marktredwitz, Germany). PyBOP was provided by Calbiochem-Novabiochem AG. Piperidine was obtained from SDS (Peypin, France); *N,N*-diisopropylethylamine (DIEA) was obtained from Merck (Darmstadt, Germany) and Triisopropylsilane (TIS) and ninhydrin were from Fluka Chemika (Buchs, Switzerland). HOAt was purchased from GL Biochem Shanghai Ltd. (Shanghai, China). Solvents for peptide synthesis and RP-HPLC (dimethylformamide (DMF), dichloromethane (DCM) and acetonitrile (MeCN)) were from Scharlau or SDS (Barcelona, Spain). Trifluoroacetic acid (TFA) was purchased from Kali Chemie (Bad Wimpfen, Germany). The other chemicals used were from Aldrich (Milwaukee, WI USA) and were of the highest purity commercially available.

### Peptide synthesis

Peptides were synthesized on a 2-Chlorotrytil chloride resin by solid-phase peptide synthesis using the 9-fluorenylmethoxycarbonyl/*tert*-butyl (Fmoc/*t*Bu) strategy. *N* ^α^-Fmoc-protected amino acids (3 eq)/HOAt (3 eq), PyBOP (4 eq), and DIEA (6 eq) were used for couplings. The Fmoc protecting group was removed by treatment with a solution of 20% piperidine in DMF. Peptides were cleaved using 2% TFA in DCM. For the cyclization step, the solvent used was DCM/DMF (98:2), PyAOP (2 eq) was dissolved in DMF and the peptide (5 mM) in DCM and once is mixed in the right proportions, 6 eq of DIEA were added, the reaction was completed in 2 or 3 h. After the cyclization was carried out, the deprotection of the side chains was performed using TFA/TIS/ H2O (95:2.5:2.5). The peptides were analysed at λ = 220 nm by analytical HPLC [Waters Alliance 2695 separation module equipped with a 2998 photodiode array detector, Sunfire C_18_ column (100 mm × 4.6 mm × 3.5 mm, 100 Å, Waters), and Empower software; flow rate = 1 mL/min. The peptides were then purified by semi-preparative HPLC [Waters 2700 Sample Manager equipped with a Waters 2487 dual λ absorbance detector, a Waters 600 controller, a Waters fraction collection II, a Symmetry C_18_ column (100 mm × 30 mm, 5 mm, 100 Å, Waters) and Millenium chromatography manager software]. Flow rate = 15 mL/min; solvents: A = 0.1% trifluoroacetic acid in water, and B = 0.05% trifluoroacetic acid in acetonitrile. Peptides were characterized by MALDI-TOF mass spectrometry (Voyager-DE RP MALDI-TOF, PE Biosystems with a N2-laser of 337 nm) and a high resolution ESI-MS model (LTQ-FT Ultra, Thermo Scientific).

### Protein

A recombinant 37 kDa polypeptide fragment containing the transmembrane and periplasmatic domains of the *E. coli* OmpA protein was purchased from NovoPro (China). OmpA samples contained Triton X-100 to mimic the membrane environment.

### Nuclear Magnetic Resonance (NMR)

All NMR experiments were recorded at 25 °C on a Bruker Avance 800 MHz spectrometer equipped with a cryoprobe. Unless otherwise is stated, AOA-2 samples (750 μM) were prepared on 10 mM sodium phosphate buffer (pH 7.3), 100% D_2_O, 0.01% NaN_3_, 1 mM Triton X-100 in the absence or in the presence of OmpA (8 μM).

Saturation transfer difference^38^ (STD) experiments were obtained using a pseudo-2D pulse sequence for the interleaved acquisition of on-resonance and off-resonance spectra. Total saturation time was 3 seconds. Suppression of the residual water signal was achieved by excitation sculpting^39^. The final STD spectrum was obtained by subtracting the on-resonance spectrum, which saturates the protein (irradiation at −0.75 ppm), from the off-resonance one (irradiation at 50 ppm), which does not affect any ligand or OmpA resonances.

Diffusion NMR experiments were performed using the LED pulse sequence^40^ with gradient lengths of 3 ms and a diffusion delay of 110 ms. The total inter-scan delay was 4.5 s. A series of experiments applying diffusion gradients of 2%, 50%, 75%, and 98% of its maximum value (55 G cm^−1^) were acquired for AOA-2 (750 μM) in 10 mM sodium phosphate buffer (pH 7.3), 100% D_2_O, 0.01% NaN_3_, 1 mM Triton X-100 in the absence and in the presence of OmpA (8 μM).

### Cell culture and infection

Human type II pneumocyte cell line A549 derived from a human lung carcinoma were obtained from American Type Culture Collection (LGC, UK) and were grown in DMEM medium (Invitrogen, Spain) supplemented with 10% heat-inactivated fetal bovine serum, vancomycin (50 μg/mL), gentamicin (20 μg/mL), amphotericin B (0.25 μg/mL) (Invitrogen, Spain) and 1% HEPES (Invitrogen, Spain) in a humidified incubator, 5% CO_2_ at 37 °C. A549 cells were routinely passaged every 3–4 days. The cells were seeded 24 h in 96-well plates for cellular viability assay, and in 24 well plates for adhesion and immnunofluorescence assays.

### Cellular viability

*Peptide toxicity*. A549 cells were incubated with AOA-1, AOA-2, AOA-3, AOA-4, AOA-5, AOA-6, or negative control peptide SXV4 (0, 0.25, 0.5 and 1 mg/mL) 48 h with 5% CO_2_ at 37 °C. Prior the evaluation of the peptides cytotoxicity, A549 cells were washed three times with prewarmed PBS. Then, peptides cytotoxicity was initially assessed quantitatively by monitoring the mitochondrial reduction activity using the MTT assay as described previously^27^. The percentage of cytotoxicity was calculated from the optical density (OD) as follow: [(OD of treated cells / mean OD of non-treated cell) × 100].

*Bacterial cytotoxicity*. A549 cells were infected with 10^8^ CFU/mL of *A. baumannii* ATCC 17978, *P. aeruginosa* PaO1, and *E. coli* ATCC 25922 strains pretreated with AOA-2 or SXV4 (0, 0.25 and 0.5 mg/mL, 30 min) for 24 h with 5% CO_2_ at 37 °C. Prior the evaluation of bacterial cytotoxicity, we firstly removed viable bacteria from A549 cells cultures and washed A549 cells five times with pre-warmed PBS. Then, cellular viability was assessed as indicated above.

### Adhesion assays

*A. baumannii* ATCC 17978, *P. aeruginosa* PaO1, and *E. coli* ATCC 25922 strains were incubated with AOA-1, AOA-2, AOA-3, AOA-4, AOA-5, AOA-6, or SXV4 (0, 0.25, 0.5 and 1 mg/mL, 30 min), and added to A549 cells for 2 h at 5% CO_2_ and 37 °C. Subsequently, infected A549 cells were washed five times with pre-warmed PBS and lysed with 0.5% Triton X-100. Diluted lysates were plated onto blood agar (Blood-Agar Columbia, Becton Dickinson Microbiology Systems, USA) and incubated at 37 °C for 24 h for enumeration of developed colonies and then the determination of the number of bacteria that attached to A549 cells.

### Immunofluorescence

The A549 cells plated on coverslips were incubated with *A. baumannii* ATCC 17978, *P. aeruginosa* PaO1 or *E. coli* ATCC 25922 strains for 2 h, and later were incubated with AOA-2 or SXV4 (0 and 0.25 mg/mL, 30 min) at 5% CO_2_ and 37 °C. Bacterial cells were removed and A549 cells were washed five times with cold PBS. A549 cells on the coverslips were fixed in methanol for 8 min at −20 °C, permeabilized with 0.5% Triton X-100 and blocked with 20% pork serum in PBS. Primary antibodies: anti-OMPs of *A. baumannii* produced in mouse kindly gift by MJ McConnell, mouse anti-*P. aeruginosa* (Abcam, Spain), mouse anti-*E. coli* (Abcam, Spain), and rabbit anti-human fibronectin (Sigma, Spain) were used at dilution of 1:50, 1:100, 1:100 and 1:400, respectively, in PBS containing 1% bovine serum albumin (BSA) for 2 h. After washing with PBS, the coverslips were incubated with their respective secondary antibodies: Alexa488-conjugated goat anti-mouse IgG, and Alexa594-conjugated goat anti-rabbit IgG (Invitrogen, Spain) at dilution of 1:100, 1:200, 1:200 and 1:800, respectively, in PBS containing 1% BSA for 1 h. The fixed coverslips were incubated for 10 min at room temperature with DAPI (Applichem, Germany) (0.5 μg/mL), washed with PBS, mounted in fluorescence mounting medium (DakoCytomation, Spain), and visualized using a Leica fluorescence microscope (DM-6000; Leica Microsystems Wetzlar GmbH, Germany).

### Fibronectin-binding assay

Fibronectin-binding assay was performed as described previously^28^. Briefly, *A. baumannii* ATCC 17978, *P. aeruginosa* PaO1, and *E. coli* ATCC 25922 grown overnight at 37 °C in LB were resuspended in PBS and collected by centrifugation at 5,000 × *g* for 10 min. Bacteria were washed twice in sterile PBS, resuspended in the same sterile buffer, and incubated with AOA-2 or SXV4 (0, 0.25 and 0.5 mg/mL, 30 min). Then, 50 µL of bacterial suspension were mixed with 50 µL of PBS, added to fibronectin coated wells and incubated 2 h at room temperature for bacterial adsorption. Non-adhered bacteria were discarded and wells were washed six times with sterile PBS to remove unbound bacteria. Adherent bacteria were then collected with 125 µL of sterile PBS containing 0.5% Triton X-100. Diluted lysates were plated onto sheep blood agar and incubated at 37 °C for 24 h for enumeration of developed colonies and then the determination of the number of bacteria that attached to fibronectin.

### Biofilm

An abiotic solid surface biofilm formation assay was performed as described previously^27^. In brief, we used an overnight culture of 2 reference strains and 8 clinical isolates of *A. baumannii*, 1 reference strain and 8 clinical isolates of *P. aeruginosa*, and 1 reference strain and 6 clinical isolates of *E. coli* diluted 1:1 in fresh Luria Bertani (LB) in 96 well plates that were incubated in presence or not of 0.25 mg/mL AOA-2 without shaking at 37 °C during 24 h. Biofilm was stained with crystal violet 0.4% (v/v) and quantified at 580 nm after solubilization with ethanol 95%.

### Animals

Immunocompetent C57BL/6 female mice (16–18 g) were obtained from University of Seville facility; they had a sanitary status of murine pathogen free and were assessed for genetic authenticity. Animals were housed in regulation cages with food and water ad libitum. This study was carried out in strict accordance with the recommendations in the Guide for the Care and Use of Laboratory Animals^41^. The protocol was approved by the Committee on the Ethics of Animal Experiments of the University Hospital of Virgen del Rocío, Seville (2012PI/238). All surgery was performed under sodium thiopental anaesthesia, and all efforts were made to minimize suffering.

### AOA-2 *in vivo* toxicity

The Reed and Munch method was used^42^. Groups of 6 mice were intraperitoneally (i.p.) inoculated with a single dose of AOA-2, starting at 10 mg/kg, in 0.5 mL NaCl 0.9%, and the solution were serially duplicated until 100% mortality was reached. The maximum tolerated dose (LD0), the LD50, and LD100 were defined as the concentrations causing 0, 50, and 100% mortality, respectively. LD50 value was determined using the Probit method.

### AOA-2 pharmacokinetics

Serum AOA-2 level was determined in healthy mice after a single i.p. administration of 10 mg/kg AOA-2. After 5, 15, 30, 60,120, 240, 480, and 1440 min, blood was extracted from the periorbital plexuses of the anesthetized mice; three mice were used for each time point. The AOA-2 level was determined using a HPLC-tandem mass spectrometry (LC-MS/MS). The maximum concentration in serum (Cmax; reported in mg per liter), the area under the concentration-time curve from time zero to ∞ (AUC_0–∞_; reported in mg-min per liter), and the terminal half-life (t_1/2_; reported in min) were calculated using a computer-assisted method^43^.

### *A. baumannii*, *P. aeruginosa* and *E. coli* peritoneal sepsis models

Murine peritoneal sepsis models with *A. baumannii* ATCC 17978, *P. aeruginosa* PaO1 or *E. coli* ATCC 25922 strains were established by i.p. inoculation of the bacteria^44^. Briefly, mice were inoculated with 0.5 mL of the bacterial suspensions, which were incubated for 20–24 h in LB at 37 °C and mixed in a 1:1 ration with a saline solution containing 10% (w/v) porcine mucin (Sigma, Spain). The minimal bacterial lethal dose 100 (MLD100), LD50 and LD0 were determined by inoculating various groups of mice (6 mice per group) with decreasing amounts of *A. baumannii* ATCC 17978, *P. aeruginosa* PaO1 and *E. coli* ATCC 25922 strains inocula from 8.5 to 2.3, 9.11 to 3.3, 9.13 to 3.47 log CFU/mL, respectively, and monitoring the survival of the mice for 7 days.

### Therapeutic effect of AOA-2 in murine models of peritoneal sepsis

The murine peritoneal sepsis models by *A. baumannii* ATCC 17978, *P. aeruginosa* PaO1 or *E. coli* ATCC 25922 strains were established by i.p. inoculation of the bacteria. Briefly, animals were infected i.p. with 0.5 mL of the MLD100 of *A. baumannii* ATCC 17978, *P. aeruginosa* PaO1, or *E. coli* ATCC 25922 strains mixed 1:1 with 10% porcine mucin. AOA-2 therapy was administered 2 h after bacterial inoculation. Fifthy-six mice were randomly ascribed to the following groups: 1). controls (without treatment), and 2). AOA-2 administered at 10 mg/kg/d i.p. 2 h after bacterial inoculation with each strain. Mortality was recorded over 72 h. After death or sacrifice of the mice at the end of the experimental period, aseptic thoracotomies were performed, and blood samples were obtained by cardiac puncture for qualitative blood cultures. The spleen and lungs were aseptically removed and homogenized (Stomacher 80; Tekmar Co., USA) in 2 mL of sterile NaCl 0.9% solution. Ten-fold dilutions of the homogenized spleen and lungs were plated onto Sheep blood agar for quantitative cultures (log CFU/g of spleen or lungs).

### Statistical analysis

Group data are presented as means ± standard errors of the means (SEM). For *in vitro* studies, the Student t test was used to determine differences between means. Differences in bacterial spleen and lung concentrations (mean ± SEM log CFU per gram of tissue) were assessed by analysis of variance (ANOVA) and *post-hoc* Dunnett’s and Tukey’s tests. Differences in mortality (%) and blood sterility (%) between groups were compared by use of the χ2 test. P values of <0.05 were considered significant. The SPSS (version 17.0; SPSS Inc.) statistical package was used.

## Electronic supplementary material

Supplementary Information 
