# Supplementary material for: Combating virulence of Gram-negative bacilli by OmpA inhibition
Source: Sci Rep. 2017 Oct 31;7:14683. doi: 10.1038/s41598-017-14972-y (PMC5666006; doi:10.1038/s41598-017-14972-y)
Supplement: Supplementary file 1 — Supplementary Information [file 41598_2017_14972_MOESM1_ESM.pdf]

Supplementary data

Title. Combating virulence of Gram-negative bacilli by OmpA inhibition.

Authors. Xavier Vila-Farrés[†1,3], Raquel Parra-Millán[†2], Viviana Sánchez-Encinales[2], Monica Varese[1], Rafael Ayerbe-Algaba[2], Nuria Bayó[1], Salvador Guardiola, María Eugenia Pachón-Ibáñez[2], Martin Kotev[1], Jesús García, Meritxell Teixidó[1], Jordi Vila[3], Jerónimo Pachón[2#], Ernest Giralt[1,4], Younes Smani[2#].

Affiliation. [1]Institute for Research in Biomedicine (IRB Barcelona), Barcelona Institute for Science and Technology (BIST), Barcelona, Spain. [2]Institute of Biomedicine of Seville (IBiS), University Hospital Virgen del Rocío/CSIC/University of Seville, Seville, Spain. [3]Barcelona Centre for International Health Research (CRESIB, Hospital Clínic-Universitat de Barcelona), Barcelona, Spain. [4]Faculty of Chemistry, University of Barcelona, Barcelona, Spain.

[†]The authors contributed equally.

Corresponding authors. [#]Jerónimo Pachón, Clinic Unit of Infectious Diseases, Microbiology and Preventive Medicine, Institute of Biomedicine of Seville (IBiS), University Hospital Virgen del Rocío, Av. Manuel Siurot s/n, 41013, Seville, Spain. Tel: +34-955923104, E-mail: pachon@us.es.

[#]Younes Smani, Clinic Unit of Infectious Diseases, Microbiology and Preventive Medicine, Institute of Biomedicine of Seville (IBiS), University Hospital Virgen del Rocío, Av. Manuel Siurot s/n, 41013, Seville, Spain. Tel: +34-955923100, E-mail: y_smani@hotmail.com, ysmani-ibis@us.es.

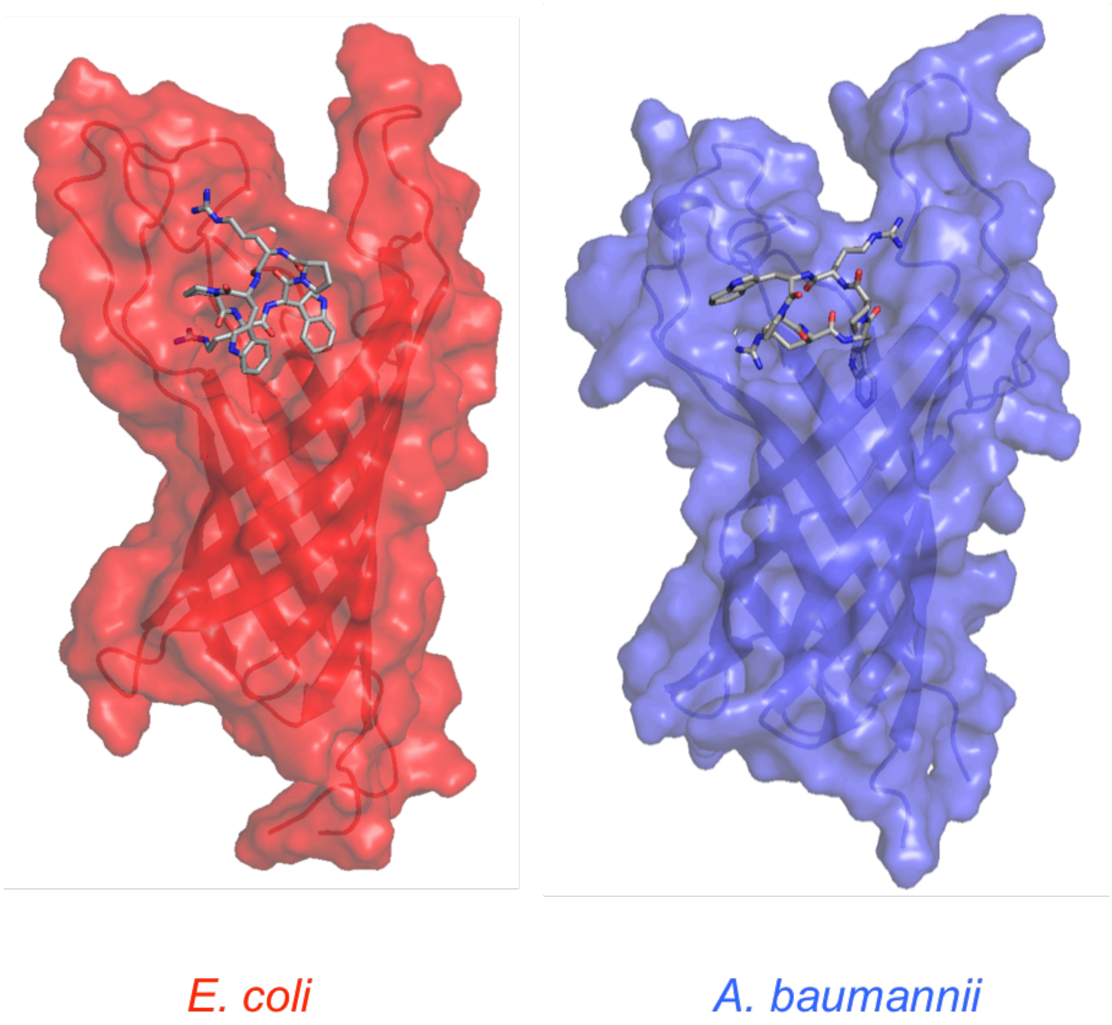

Figure S1. Structural models generated by docking of peptide AOA-2 into the NMR solution structure of the transmembrane domain of *E. coli* OmpA (PDB 1G90) and into the homology model of *A. baumannii* OmpA generated as described under "Methods". AOA-2 is displayed as sticks.

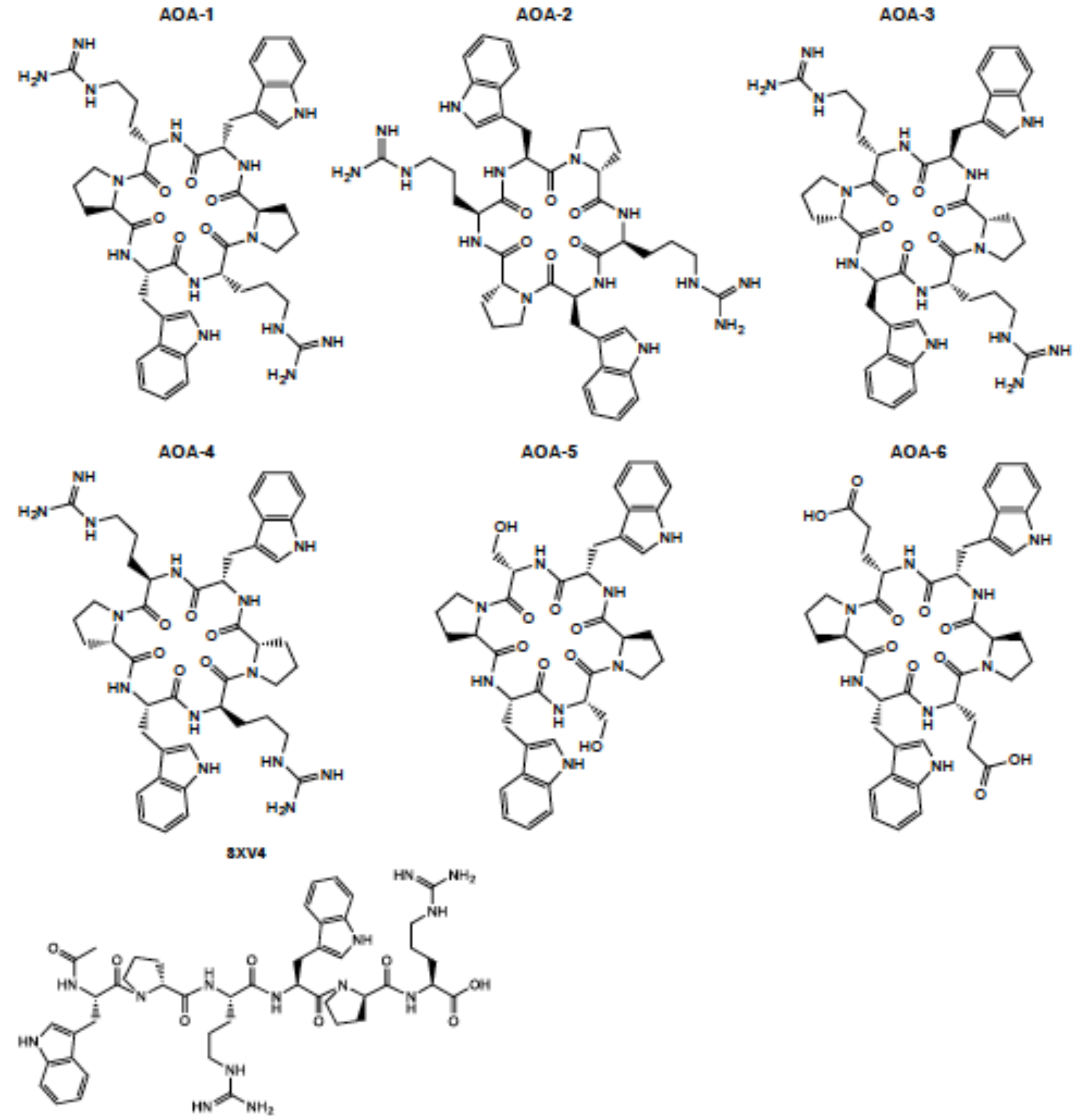

Figure S2. Structure of six cyclic hexapeptides and peptide control (SXV4) used in the study.

Table S1. MICs of the six hexapeptides for *A. baumannii* and *E. coli*.

**Peptides**	**MIC (µg/mL)**		
	***A. baumannii* ATCC 17978**	***P. aeruginosa* PaO1**	***E. coli* ATCC 25922**
AOA-1	>500	>500	>500
AOA-2	>500	>500	>500
AOA-3	>500	>500	>500
AOA-4	>500	>500	>500
AOA-5	>500	>500	>500
AOA-6	>500	>500	>500
SXV4	>500	>500	>500

MIC: minimal inhibitory concentration

Table S2. Percentages of A549 cellular viability assessed through mitochondrial reduction activity (MTT assay) in presence of anti-OmpA hexapeptides and control peptides (SXV4 and COL). COL: colistin. ND: not determined.

	Cellular viability (%)			
**Peptides**	**0.25 mg/mL**	**0.5 mg/mL**	**1 mg/mL**	**5 mg/mL**
AOA-1	98.89 ± 0.41	99.69 ± 0.26	99.7 ± 0.38	ND
AOA-2	98.09 ± 0.27	98.33 ± 0.4	98.48 ± 0.55	ND
AOA-3	99.27 ± 0.21	99.7 ± 0.14	99.36 ± 0.3	ND
AOA-4	99.47 ± 0.39	99.77 ± 0.17	100.13 ± 0.18	ND
AOA-5	98.86 ± 0.08	100.05 ± 0.39	100.14 ± 0.46	ND
AOA-6	98.31 ± 0.35	99.14 ± 0.44	99.38 ± 0.34	ND
SXV4	98.17 ± 0.6	98.79 ± 0.28	98.55 ± 0.21	ND
COL	ND	91.85 ± 2.67	68.39 ± 2.93	9.88 ± 0.42

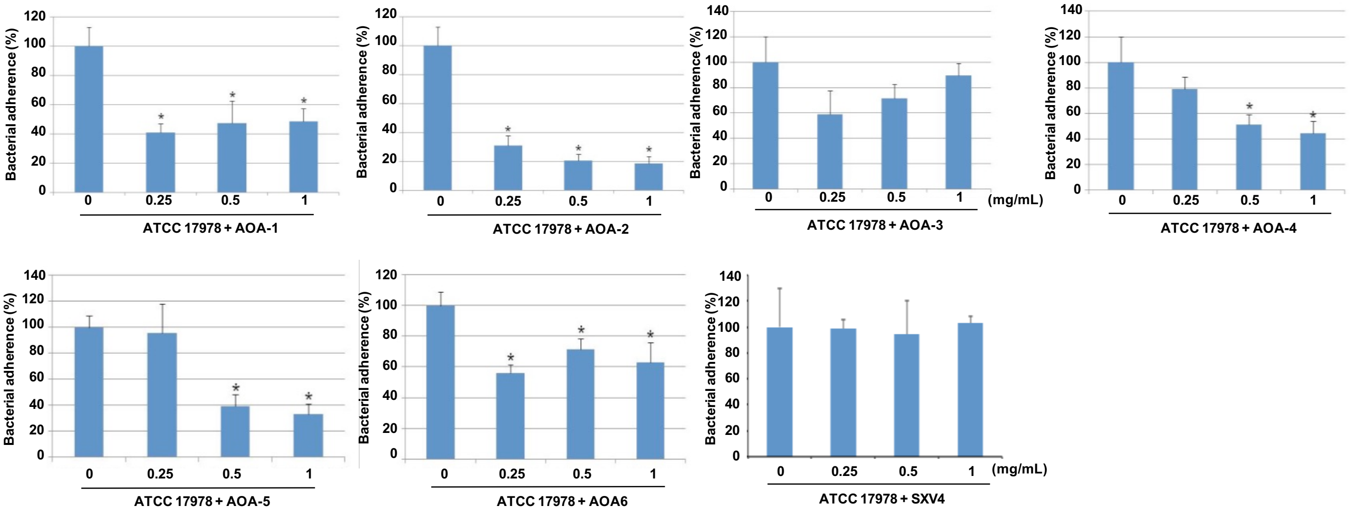

Figure S3. OmpA inhibitor hexapeptides reduce *A. baumannii* adherence to A549 cells. A549 cells were infected with 10^8 CFU/mL of ATCC 17978 strain during 2 h in presence of AOA-1, AOA-2, AOA-3, AOA-4, AOA-5, AOA-6, or SXV4 (0, 0.25, 0.5, or 1 mg/mL). The percentage of bacterial adherence was calculated as follow: [(number of colonies of treated A549 cells with peptide / number of colonies of non-treated cells) x 100]. Representative results of three independent experiments are shown; data are means ± SEM. *P<0.05: between untreated and treated groups.

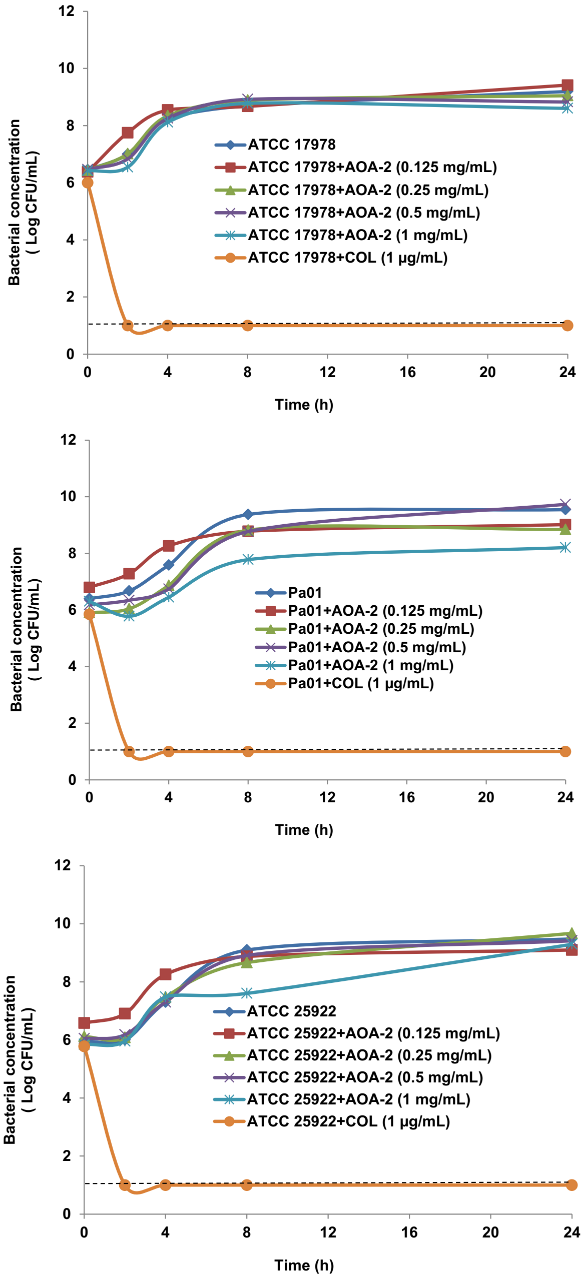

Figure S4. Time kill curves of *A. baumannii* ATCC 17978, *P. aeruginosa* PaO1, and *E. coli* ATCC 25922 strains in presence of 0.125, 0.25, 0.5 and 1 mg/mL AOA-2, and 1 μg/mL colistin. COL: colistin.

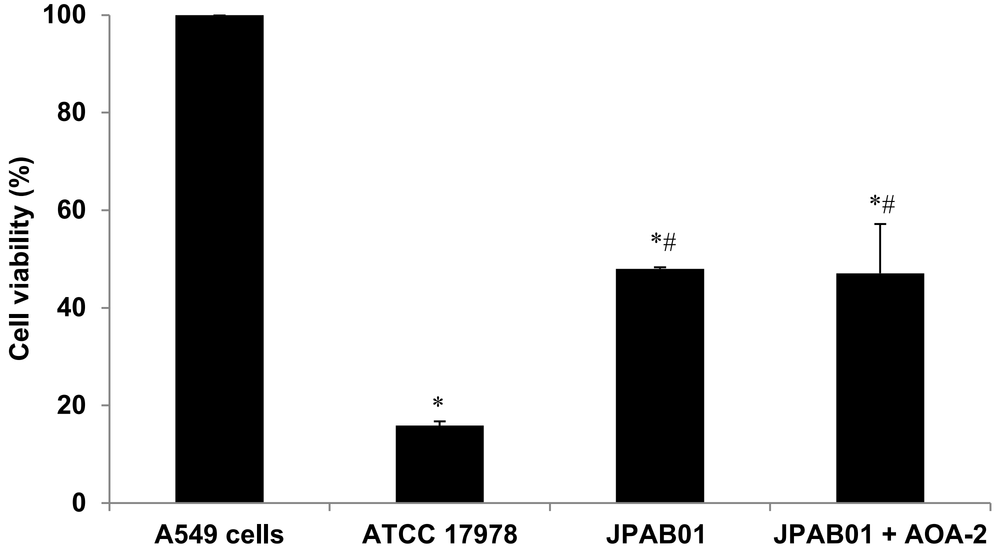

Figure S5. Cell death induced by *A. baumannii* ATCC 17978 and JPAB01 strains. A549 cells were infected for 48 h with 10^8 CFU/mL of ATCC 17978 and JPAB01 strains treated with AOA-2 (0 or 0.5 mg/mL). Bacterial cytotoxicity was assessed by monitoring the mitochondrial reduction activity using the MTT assay. Representative results of two independent experiments are shown; data are means ± SEM. JPAB01: *ompA*-deficient ATCC 17978 strain. *P<0.05: between infected and non-infected cells, $^{\#}P$<0.05: between untreated and treated groups.

Table S3. *In vivo* toxicity of AOA-2. Each group of six mice received 0.5 ml of AOA-2 at 10, 20, 40, 80 or 160 mg/kg. Mice survival was monitored for a one week and lethal doses (LD) 0, 50 and 100 were calculated.

	**LD0**	**LD50**	**LD100**
**AOA-2 (mg/kg)**	40	85	160

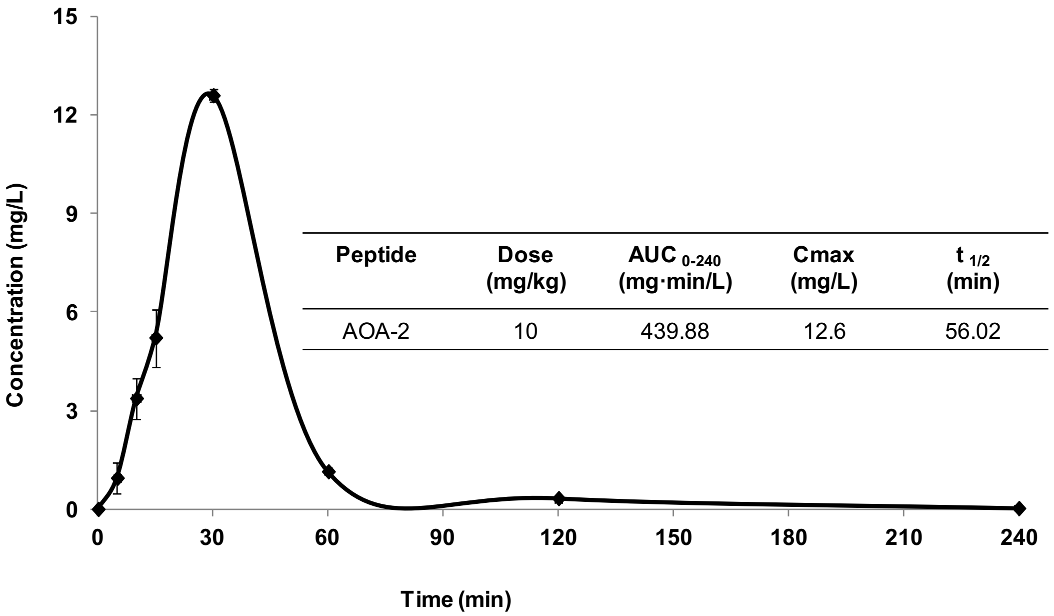

Peptide	Dose (mg/kg)	AUC 0-240 (mg·min/L)	Cmax (mg/L)	t 1/2 (min)
AOA-2	10	439.88	12.6	56.02

Figure S6. Serum AOA-2 pharmacokinetic parameters. AOA-2 was intraperitoneally administered to mice at a dose of 10 mg/kg, and serum AOA-2 concentrations were analyzed for 240 min. C_{max}, maximum concentration in serum; $t_{1/2}$, elimination half-time; $AUC_{0\text{-}240}$, area under the concentration-time curve from time 0 to 240 min.

Table S4. Bacterial strains, plasmids, and primers used in this study.

Strains	Relevant features and use	References
A. baumannii ATCC 17978 wt	Reference strain isolated in a fatal meningitis infant	1
A. baumannii ATCC 19606	Reference strain isolated from urine, United States, 1948.	2
A. baumannii C4, C5, C12	Colonizing MDR strains isolated from tracheobronchial aspirate of mechanically ventilated individual adult patients	3
A. baumannii IB1, IB2	Bacteremic MDR strains isolated from tracheobronchial aspirate of mechanically ventilated individual adult patients	3
A. baumannii HC1, HC2	Bacteremic MDR strains isolated from blood of mechanically ventilated individual adult patients	3
A. baumannii 77	MDR strain isolated from respiratory sample.	4
P. aeruginosa PaO1	Reference strain	5
P. aeruginosa 15, 160	Bacteremic MDR strains isolated from blood	6
P. aeruginosa 17, 61, 127, 142, 184, 204	Bacteremic strains isolated from blood	6
E. coli ATCC 25922	Reference strain. Serotype O6, Biotype 1	7
E. coli 12-69, 7-9, 7-2, 11-51-2, 5-38, 12-74	Bacteremic strains isolated from blood in the Virgen Macarena University hospital of Seville.	--

References

1. Baumann, P., Doudoroff, M. & Stanier, R.Y. A study of the Moraxella group. II. Oxidative-negative species (genus Acinetobacter). *J. Bacteriol.* **95**, 1520-1541 (1968).
2. Schaub, I.G. & Hauber, F.D. A biochemical and serological study of a group of identical unidentifiable gram-negative bacilli from human sources. *J. Bacteriol.* **56**, 379-385 (1948).
3. Labrador Herrera, G. et al. Draft Genome sequences of seven multidrug-resistant *Acinetobacter baumannii* strains, isolated from respiratory samples in Spain. *Genome Announc.* **4**: e00083-16 (2016).
4. Smani, Y. et al. Platelet-activating factor receptor initiates contact of *Acinetobacter baumannii* expressing phosphorylcholine with host cells. *J. Biol. Chem.* **287**, 26901-26910 (2012).
5. Holloway, B.W. Genetic recombination in *Pseudomonas aeruginosa*. *J. Gen. Microbiol.* **13**, 572-581 (1955).
6. Peña, C. et al. Prospective multicenter study of the impact of carbapenem resistance on mortality in *Pseudomonas aeruginosa* bloodstream infections. *Antimicrob. Agents Chemother.* 56, 1265-1272 (2012).
7. Boyle, V.J., Fancher M.E. & Ross, R.W. Jr. Rapid, modified Kirby-Bauer susceptibility test with single, high-concentration antimicrobial disks. *Antimicrob. Agents Chemother.* **3**, 418-424 (1973).
